# High Pressure Crystal Structure and Electrical Properties of a Single Component Molecular Crystal [Ni(dddt)_2_] (dddt = 5,6-dihydro-1,4-dithiin-2,3-dithiolate)

**DOI:** 10.3390/molecules24101843

**Published:** 2019-05-14

**Authors:** Hengbo Cui, Takao Tsumuraya, Hamish H.-M. Yeung, Chloe S. Coates, Mark R. Warren, Reizo Kato

**Affiliations:** 1 Condensed Molecular Materials Laboratory RIKEN, 2-1 Hirosawa, Wako-shi, Saitama 351-0198, Japan; reizo@riken.jp; 2 Priority Organization for lnnovation and Excellence (POIE), Kumamoto University, 2-39-1 Kurokami, Kumamoto 860-8555, Japan; tsumu@kumamoto-u.ac.jp; 3 Inorganic Chemistry Laboratory, University of Oxford, South Parks Road, Oxford OX1 3QR, UK; chloe.coates@chem.ox.ac.uk; 4 Beamline I19, Diamond Light Source, Harwell Campus, Didcot OX11 0DE, UK; mark.warren@diamond.ac.uk

**Keywords:** single-component, molecular conductor, high pressure, resistivity, X-ray diffraction, density functional theory, pressure-dependent, diamond anvil cell, synchrotron, semiconductor

## Abstract

Single-component molecular conductors form an important class of materials showing exotic quantum phenomena, owing to the range of behavior they exhibit under physical stimuli. We report the effect of high pressure on the electrical properties and crystal structure of the single-component crystal [Ni(dddt)_2_] (where dddt = 5,6-dihydro-1,4-dithiin-2,3-dithiolate). The system is isoelectronic and isostructural with [Pd(dddt)_2_], which is the first example of a single-component molecular crystal that exhibits nodal line semimetallic behavior under high pressure. Systematic high pressure four-probe electrical resistivity measurements were performed up to 21.6 GPa, using a Diamond Anvil Cell (DAC), and high pressure single crystal synchrotron X-ray diffraction was performed up to 11.2 GPa. We found that [Ni(dddt)_2_] initially exhibits a decrease of resistivity upon increasing pressure but, unlike [Pd(dddt)_2_], it shows pressure-independent semiconductivity above 9.5 GPa. This correlates with decreasing changes in the unit cell parameters and intermolecular interactions, most notably the *π*-*π* stacking distance within chains of [Ni(dddt)_2_] molecules. Using first-principles density functional theory (DFT) calculations, based on the experimentally-determined crystal structures, we confirm that the band gap decreases with increasing pressure. Thus, we have been able to rationalize the electrical behavior of [Ni(dddt)_2_] in the pressure-dependent regime, and suggest possible explanations for its pressure-independent behavior at higher pressures.

## 1. Introduction

In the development of single-component molecular conductors, metal dithiolene complexes, with a small energy gap between the highest occupied molecular orbital (HOMO) and the lowest unoccupied molecular orbital (LUMO), have formed an important category. The conductivity of such complexes can be greatly enhanced by changing the HOMO-LUMO gap, via increased *π* conjugation and flatness of the ligand part. For example, as-synthesized [M(tmdt)_2_] (tmdt = trimethylenetetrathiafulvalenedithiolate, M = Ni, Au) single crystals exhibit metallic behavior [1,2].

At ambient pressure, most neutral single-component metal dithiolene crystals are semiconducting, or electrically insulating. However, owing to weak intermolecular interactions, molecular crystals have soft lattices. Therefore, the application of pressure to insulating phases is an efficient way to reduce intermolecular distances, without altering intramolecular geometries substantially, leading to the discovery of new single-component molecular metals. Indeed, we have modified the four-probe resistivity measurement technique, by using a Diamond Anvil Cell (DAC), and successfully found that insulating [Ni(ptdt)_2_] (ptdt = propylenedithiotetrathiafulva-lenedithiolate) [3] and [Cu(dmdt)_2_] (dmdt = dimethyltetrathiafulvalenedithiolate) [4] turn metallic at 19.4 GPa and 4.7 GPa, respectively. Recently, we also discovered the first single-component molecular superconductor, based on a metal dithiolene complex, [Ni(hfdt)_2_] (hfdt = bis(trifluoro-methyl)tetrathiafulvalenedithiolate) at 8 GPa (*T*_c_ = 5.5 K) [5].

Chemical modifications to increase *π* conjugation and ligand flatness can also reduce the solubility of the dithiolene complex anion salt used as a starting material. As a consequence, obtaining large crystals of such single-component molecular conductors is very difficult. By comparison, smaller metal dithiolene complexes have recently attracted considerable attention, owing to their simple synthesis and ease of obtaining large single crystals. Lorcy and co-workers found that the small-sized complex [Au(Me-thiazdt)_2_] (where Me-thiazdt = N-methyl-1,3-thiazoline-2-thione-4,5-dithiolate) [6] showed metallic behavior at ambient pressure. Previous studies of its analogs, [Au(Et-thiazdt)_2_] [7] and [Au(Et-thiazds)_2_], which features S substituted by Se [8], showed that they turn metallic at 1.3 GPa and 0.6 GPa, respectively, values which compare favorably with the simple metal dithiolene complex, [Ni(dmit)_2_], which becomes metallic at 15.9 GPa [9]. In single-component molecular conductors that meet certain conditions, the HOMO band and LUMO band can touch at a single point to form a Dirac electron system [10,11]. We have employed first-principles density functional theory (DFT) calculations to understand the single-component crystal [Pd(dddt)_2_] (dddt = 5,6-dihydro-1,4-dithiin-2,3-dithiolate), which exhibits temperature-independent resistivity at 12.6 GPa. The results indicated that anisotropic Dirac cones emerge by a closing of the HOMO and LUMO bands originating from crystallographically-independent layers. Furthermore, the combined effect of the intralayer HOMO-HOMO/LUMO-LUMO and interlayer HOMO-LUMO interactions play an important role [10]. Further detailed calculations showed that, unlike the conventional molecular massless Dirac electron system *α*-(BEDT-TTF)_2_I_3_, [Pd(dddt)_2_] has a loop of the Dirac points in the 3-D Brillouin zone, rather than a line [12,13]. That is, the crossing points of linear bands (Dirac points) form a loop in reciprocal space, and such a unique electronic state is called a nodal line semimetal.

In order to investigate the effects of chemical substitution in single-component Dirac-cone materials, we synthesized and measured the high pressure resistivity of the isoelectronic complex [Ni(dddt)_2_], which was previously reported to be isostructural to [Pd(dddt)_2_] [14]. Considering the small contribution of the central transition metal *d*-orbitals to the frontier orbitals of metal dithiolene complexes, it was expected that [Ni(dddt)_2_] would show similar electronic properties to [Pd(dddt)_2_]. We sought to explain the results using experimental structural data obtained in situ for the first time from high pressure single crystal synchrotron X-ray diffraction, and first-principles band structure calculations based on the high pressure structures. In this way, we could rationalize the high pressure-induced changes in electrical properties, in particular the initial decrease in resistivity above ambient pressure, and suggest reasons for subsequent pressure-independent resistivity above 9.5 GPa.

## 2. Results

It has previously been reported that a single crystal of [Ni(dddt)_2_] could be obtained by a recrystallization of powder from benzene, which was prepared by I_2_ oxidation of [(*n*-C_4_H_9_)_4_N][Ni(dddt)_2_] [14,15]. In order to avoid using toxic benzene, we freshly prepared single crystals of [Ni(dddt)_2_] by I_2_ oxidation in acetone, using an H-shaped diffusion cell sitting on a non-vibration plate at room temperature (see Materials and Methods section). 

**Figure 1:**
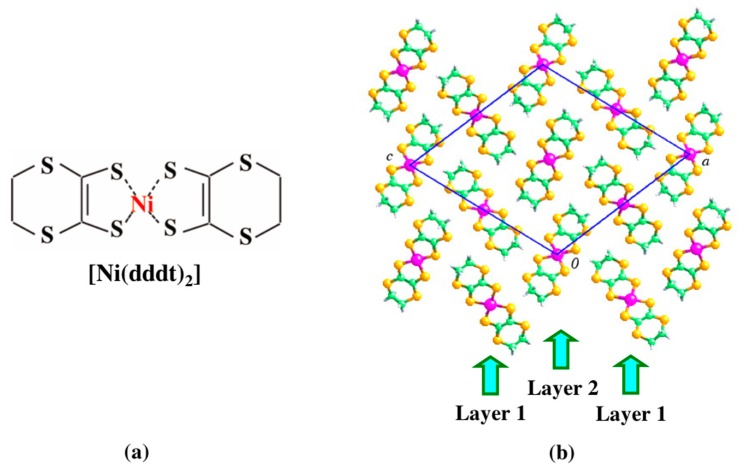
(**a**) Molecular structure of [Ni(dddt)_2_], and (**b**) its crystal structure viewed down the *b* axis, highlighting the crystallographically-independent layers 1 and 2. Ni, C, H and S are shown in magenta, green, grey and yellow, respectively.

Single crystal X-ray diffraction confirmed the crystal structure to be isostructural with [Pd(dddt)_2_], albeit with slightly smaller cell parameters (Figure 1). Crystals synthesized in this way were used for subsequent resistivity measurements and single crystal structure determination under high pressure.

### 2.1. Electrical Resistivity

**Figure 2:**
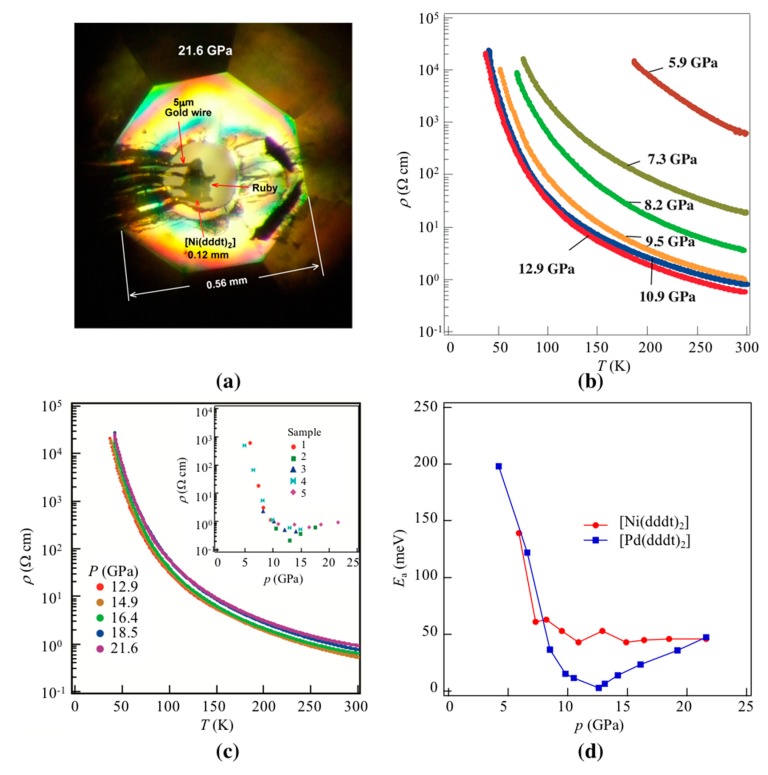
(**a**) Optical microscope image of the [Ni(dddt)_2_] sample at 21.6 GPa assembled in a Diamond Anvil Cell (DAC). (**b**,**c**) Temperature-dependent resistivity, *ρ*, under various pressures; inset in (**c**) shows the pressure dependence of room temperature resistivity. (**d**) Pressure dependence of activation energy, *E*_a_, showing data for [Pd(dddt)_2_] for comparison.

Resistivity measurements were performed by a DC four-probe Diamond Anvil Cell technique, as described previously [3], using the setup shown in Figure 2a (see Materials and Methods section for details). Figure 2b,c show the temperature dependence of resistivity under various pressures. Figure 2c, inset, shows the pressure dependence of the room temperature resistivities. At ambient pressure, resistivity was higher than our equipment range. Upon increasing the pressure, the room temperature resistivity decreased gradually, such that by 5.9 GPa, the room temperature conductivity, *σ*_rt_, was 1.6 × 10^−3^ S cm^−1^, and the activation energy, *E*_a_, was 0.14 eV. At 10.9 GPa, *σ*_rt_ was 1.2 S cm^−1^ and *E*_a_ was 0.043 eV. At higher pressures, the temperature-dependent data look very similar, indicating resistivity behavior that is independent of pressure; at 21.6 GPa, *σ*_rt_ and *E*_a_ were 1.1 S cm^−1^ and 0.046 eV, respectively. Values for *E*_a_ also show pressure independence from 7.3 GPa to 21.6 GPa (see Figure 2d). Most strikingly, the experimental data show that [Ni(dddt)_2_] remains semiconducting at all pressures measured, in contrast to [Pd(dddt)_2_].

### 2.2. High Pressure Single Crystal Structure

**Figure 3:**
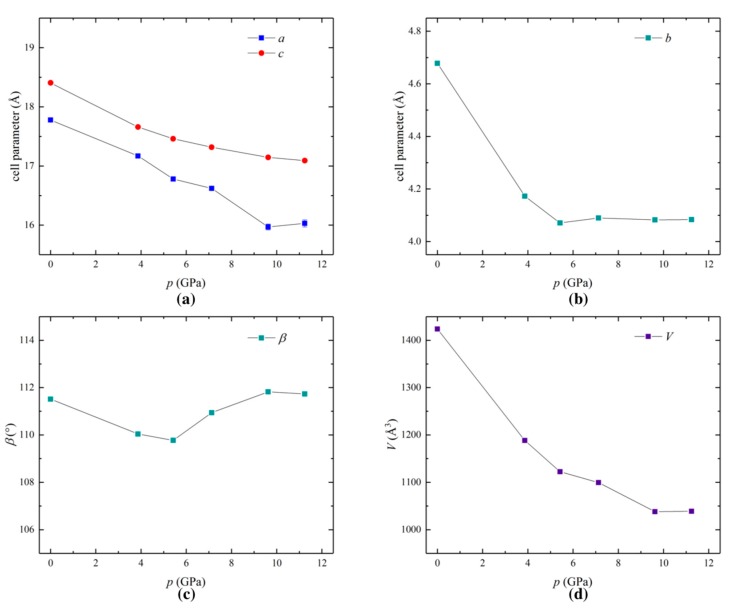
Changes in the crystallographic unit cell parameters of [Ni(dddt)_2_], showing variations in (**a**) the *a* and *c* axes, (**b**) the *b* axis, (**c**) the monoclinic angle, *β*, and (**d**) the unit cell volume, *V*.

In order to understand the origins of the high pressure electronic behavior, high pressure single crystal X-ray diffraction data were collected at beamline I19-2, Diamond Light Source synchrotron, UK, at increasing pressures of 0.1 MPa, 3.9 GPa, 5.4 GPa, 7.1 GPa, 9.6 GPa and 11.2 GPa (see Materials and Methods section for details). Figure 3 shows the changes in unit cell parameters as a function of pressure. The unit cell lengths *a* and *c* decrease reasonably smoothly from 17.777(8) Å to 16.03(6) Å and from 18.405(3) Å to 17.090(7) Å, respectively (Figure 3a). In contrast, the *b* axis, which corresponds to the molecular stacking direction, decreases sharply from 4.6782(3) Å at 0.1 MPa to 4.1726(5) Å at 3.9 GPa, before plateauing at around 4.1 Å above 5.4 GPa (Figure 3b). 

**Figure A1:**
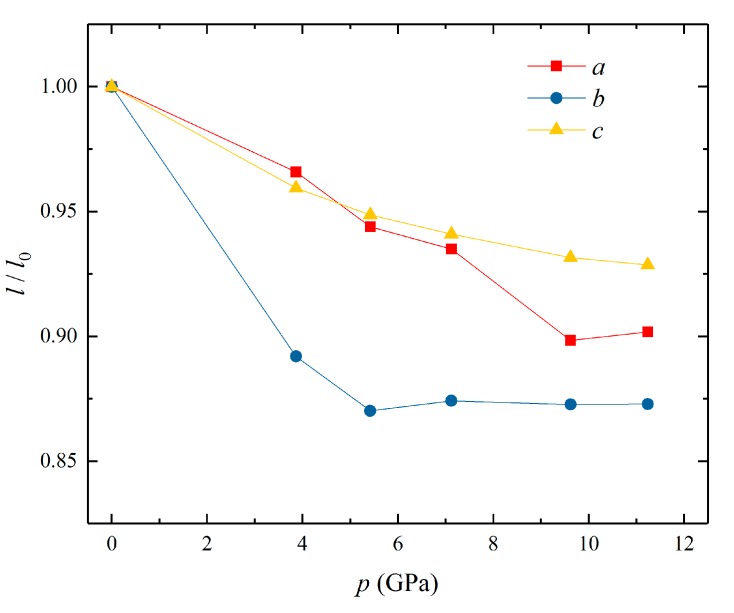
Relative changes in crystallographic lattice parameters as a function of pressure, based on experimental single crystal structure determinations. *a*, *b*, and *c* are shown as red squares, blue circles and yellow triangles, respectively.

At low pressures, *b* is by far the most compressible cell parameter: at 5.4 GPa, the length of *b* is just 0.87 times its ambient pressure value, whilst *a* and *c* are 0.94 and 0.95 times their ambient values, respectively (see Appendix A Figure A1). This is perhaps to be expected, owing to the anisotropy of the crystal lattice, and the softness of *π*-*π* interactions. The monoclinic angle, *β*, decreases from 111.51(2)° at 0.1 MPa to 109.77(4)° at 5.4 GPa, before increasing again to 111.82(7)° at 9.6 GPa (Figure 3c). Overall, the change in unit cell volume, *V*, is a fairly smooth decrease from 1424(4) Å at 0.1 MPa to 1039(3) Å^3^ at 9.6 GPa (Figure 3d).

**Figure 4:**
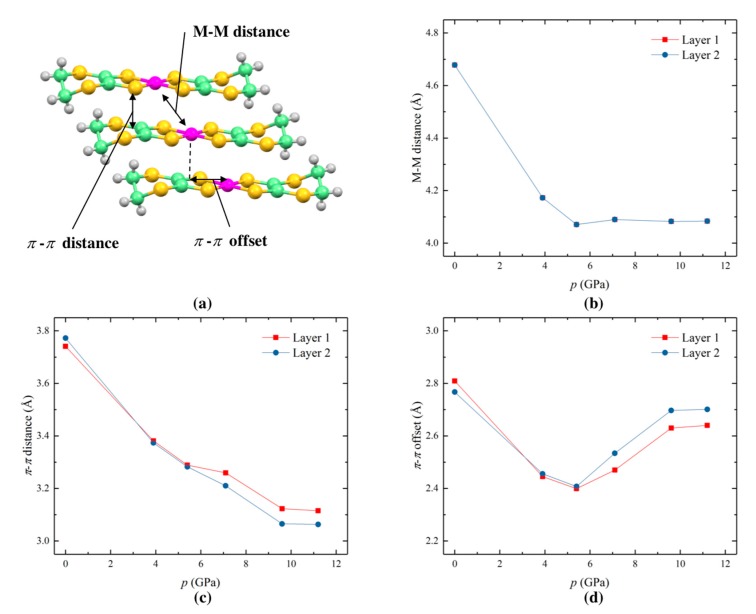
Changes in intermolecular distances in [Ni(dddt)_2_], shown in (**a**), as a function of pressure for crystallographically-independent Layer 1 and Layer 2. (**b**) Metal–metal distance (note that values for Layer 1 and Layer 2 are equivalent and therefore overlap), (**c**) *π*-*π* distance, and (**d**) *π*-*π* offset.

The limited resolution of the high pressure single crystal data means that discussion of C–C and C–S bond distances, which were restrained in order to stabilize the refinements, cannot yield meaningful insight. However, analysis of some simple intermolecular geometrical parameters gives some indication of the molecular origins of the mechanical properties (Figure 4a–d). The behavior of the two crystallographically-independent layers is very similar. Distances between the metal atoms, which fall on special positions, is fixed by symmetry, and identical to the magnitude of the *b* axis for both Layer 1 and Layer 2. In other words, the M-M distance decreases rapidly with pressure up to 5.4 GPa, and then plateaus around 4.1 Å (Figure 4b). The *π*-*π* stacking distance, defined here as the intermolecular distance between the centroid of one ligand and the plane defined by the ligand in the molecule opposite, decreases smoothly across the entire pressure range of 0.1 MPa to 11.2 GPa, from around 3.75 Å to 3.1 Å, respectively, with an apparent leveling-off above 9.6 GPa (Figure 4c). The *π*-*π* offset distance, on the other hand, decreases from around 2.8 Å at 0.1 MPa to 2.4 Å at 5.4 GPa and then increases with increasing pressure to around 2.7 Å at 11.2 GPa.

### 2.3. High Pressure DFT Calculations

In order to connect the experimentally-observed high pressure resistivity data with the structural information provided by high pressure single crystal synchrotron X-ray diffraction, first-principles DFT band structure calculations were performed, based on the experimental unit cell parameters and atomic coordinates (see Materials and Methods section).

**Figure 5:**
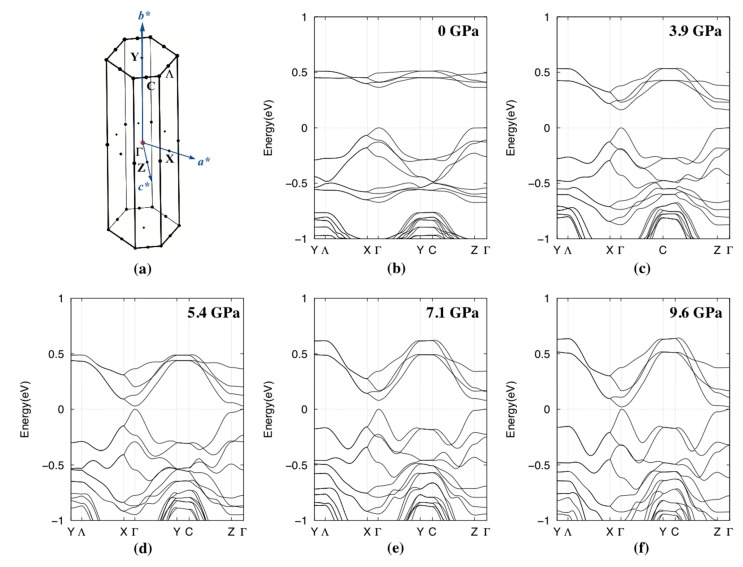
High pressure electronic band structures calculated with the GGA-PBE functional, based on the experimentally-determined structures of [Ni(dddt)_2_]: (**a**) Brillouin zone and the reciprocal lattice vectors of the structure in *P*2_1_/*a*, (**b**) ambient pressure, (**c**) 3.9 GPa, (**d**) 5.4 GPa, (**e**) 7.1 GPa, and (**f**) 9.6 GPa. The dotted line at Energy = 0 eV represents the top of the valence bands.

**Figure A2:**
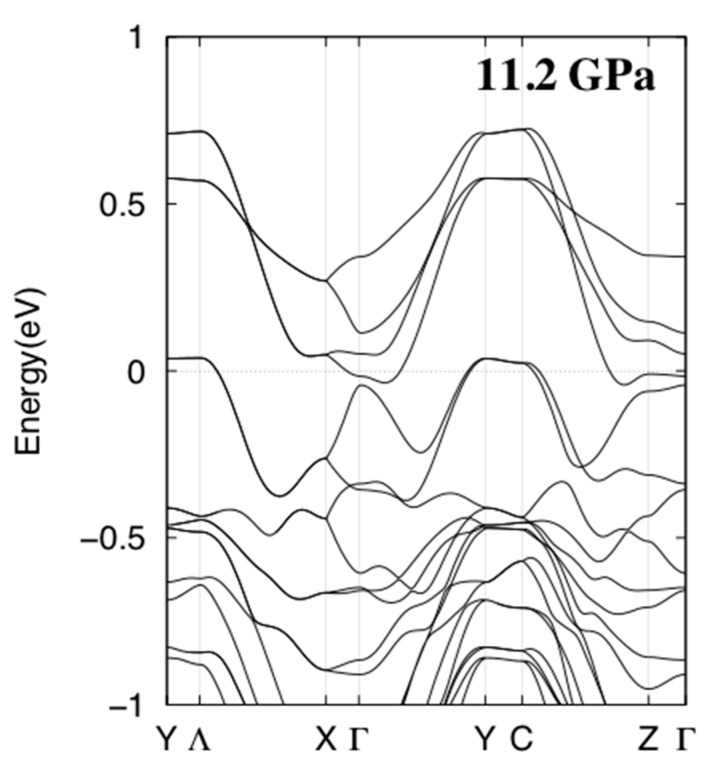
Band structure calculated from first principles DFT for the experimentally-determined structure at 11.2 GPa.

Overall, the effect of pressure is to broaden the bands of the frontier molecular orbitals, such that the band gap decreases (Figure 5). At low pressures, the band gap is direct, and is the smallest at the Γ point; in particular, the LUMO band decreases in energy near the Γ point, such that the band gap changes from 0.37 eV at 0 GPa to 0.16 eV at 3.9 GPa, 0.03 eV at 5.4 GPa, 0.08 eV at 7.1 GPa, and 0.02 eV at 9.6 GPa. At 11.2 GPa, the bands distort sufficiently to overlap, suggesting semi-metal character (see Appendix A Figure A2). This result contrasts somewhat with the experimental resistivity measurements, which indicate semiconducting behavior at all pressures, and may be due to discrepancies in the quality of hydrostatic pressure between resistivity measurements and X-ray diffraction data collection. Daphne oil 7373, which has a hydrostatic limit around 2.2 GPa, was used for the former [16], whilst 4:1 methanol:ethanol, which has a hydrostatic limit of 9.8–10.5 GPa, was used for the latter [17]. Alternatively, the difference between the calculated semi-metallic band structure and insulating resistivity at the high pressure range (>11 GPa) may come from the problem of band gap underestimation in the GGA calculations. We will discuss this aspect in a forthcoming paper, together with the results by the hybrid functional HSE06 [18,19].

## 3. Discussion

The DC four-probe resistivity measurements show that the single-component molecular crystal [Ni(dddt)_2_] exhibits a semiconducting behavior at high pressure between 0.1 MPa and 21.6 GPa. Resistivity and activation energy decrease as a function of increasing pressure up to around 10.9 GPa, above which they are pressure independent. This behavior contrasts with the isoelectronic, isostructural system [Pd(dddt)_2_], which exhibits temperature-dependent resistivity, metallization and formation of Dirac cones in the electronic band structure at 12.6 GPa.

High pressure crystal structures, obtained using single crystal synchrotron X-ray diffraction, show that unit cell lengths *a* and *c*, and volume, *V*, decrease with increasing pressure, and become less compressible towards 11.2 GPa, the limit of our structure determination measurements. While this correlates with the range of pressure-dependent resistivity, and is suggestive of a stiffening of the crystal lattice, the lack of data in the pressure-independent resistivity region prohibits firm conclusions as to its direct cause. Interestingly, the crystallographic *b* axis, which corresponds directly to the molecular stacking direction and the intralayer Ni–Ni distance, plateaus much earlier, around 5.4 GPa. It alone cannot be directly responsible for the electronic behavior. Indeed, the *π*-*π* stacking distance decreases over the entire pressure range in a similar fashion to *a*, *c* and *V*. The changes in *π*-*π* offset distance bear more similarity to the changes in monoclinic angle, *β*, and are likely to be a consequence of subtle variations in intra-chain electrostatic interactions as a function of *π*-*π* stacking distance. Therefore, the structural features most important to the electronic behavior are likely to be the *π*-*π* stacking distance and inter-chain interactions, the latter of which are outside the scope of this analysis.

Electronic band structures, calculated using DFT at various pressures up to 11.2 GPa, show that the band gap decreases with increasing pressure, which is in general agreement with the decrease in activation energy found by the resistivity measurements (Table 1). As pressure increases up to 9.6 GPa, the frontier orbital bands broaden, leading to a decreasing direct band gap at the Γ point. The slight increase in calculated band gap at 7.1 GPa correlates to small increases in the M-M distance (Figure 4b) and *π*-*π* offset (Figure 4d); however, it should be noted that the refinement indicators at this pressure were above average, even for high pressure datasets (wR_gt 0.36, R_all 0.15). Further broadening at 11.2 GPa, in particular Y and M points of the HOMO bands, results in the apparent indirect overlap of both the HOMO and LUMO bands. This would suggest semi-metal behavior, which was not observed in the experimental measurements. One possible explanation for this difference is the uncertainty of atomic coordinates in the experimental crystal structure, upon which the DFT calculation was based, owing to being above the hydrostatic limit of the pressure-transmitting fluid. It is known that small changes in intermolecular interactions, resulting from subtle changes in *π*-*π* stacking and offset, can cause large discrepancies between calculated and experimental data.

Overall, we find that the changes in electrical resistivity correlate well with crystal structure changes and DFT calculations from ambient pressure to around 10 GPa. As the *π*-*π* stacking distance decreases, the measured resistivity, activation energy and calculated band gaps all decrease. The origins of pressure independence in the resistivity measurements above 10.9 GPa are most likely the reduced changes in compressed crystal structure, but any discrepancy between different analyses and a lack of experimental structural data leaves this an open question for future investigation.

## 4. Materials and Methods

### 4.1. Synthesis and Ambient Pressure Crystallography

Single crystals of [Ni(dddt)_2_] were prepared by I_2_ oxidation in acetone using an H-shaped diffusion cell sitting on a non-vibration plate at room temperature. One side of the cell was set with [(*n*-C_4_H_9_)_4_N][Ni(dddt)_2_] (16.9 mg), and the other side was set with I_2_ (52 mg) and acetic acid (2.5 mL), and then filled with acetone (15 mL). Black needle-shaped single crystals were obtained after about 10 days. Crystal data as follows: [Ni(dddt)_2_] C_8_H_8_NiS_8_, M 419.37, monoclinic, space group *P*2_1_/*n* (no. 14), *a* = 17.805(3) Å, *b* = 4.6626(6) Å, *c* = 18.337(3) Å, *β* = 111.745(18)°, *V* = 1413.9(4) Å^3^, *Z* = 4.

### 4.2. High Pressure Electrical Resistivity

Four 5 μm gold wires were attached to the sample with gold paint, and placed in the sample chamber of the Diamond Anvil Cell (DAC), with a culet size of 0.56 mm. Inconel 625 was used as the metal gasket, and Daphne Oil 7373 was used as the pressure medium. The pressure was determined by the shift of Ruby fluorescence R1 lines at room temperature. At each pressure point, DC resistivity was measured at temperatures below 300 K, decreasing until the experimental limit was reached.

### 4.3. High Pressure Single Crystal Structure Determination

**Figure A3:**
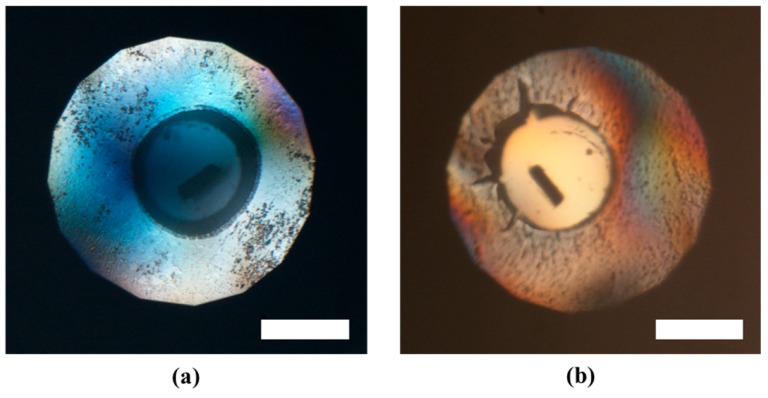
Optical microscope images of the sample studied by high pressure single crystal diffraction (**a**) prior to measurement at 9.6 GPa, front view, and (**b**) post measurement at 11.2 GPa, reverse view (scale bar = 200 μm).

High pressure single crystal synchrotron X-ray diffraction data were collected on Beamline I19-2 Diamond Light Source, UK using 0.4859 Å radiation. A crystal of [Ni(dddt)_2_] was placed in a DAC with a culet size of 0.6 mm and a tungsten gasket. 4:1 methanol:ethanol was used as the hydrostatic pressure medium, and the pressure was determined by the shift of Ruby fluorescence R1 lines at room temperature, before and after each measurement. Optical microscope images of the sample prior to measurement at 9.6 GPa, and after measurement at 11.2 GPa, are shown in Appendix A Figure A3. Data were processed via in-house semi-automated routines using aimless [20], ccp4 [21], dials [22], pointless [23], and xia2 [24], and solved and refined using SHELX [25] within Olex2 [26]. Owing to the low completeness of the high pressure datasets, C–C and C–S distances were restrained, and CH_2_ hydrogen positions were refined with riding coordinates. Crystallographic information files are available from the CCDC, reference numbers 1908148–1908153 (see Supplementary Materials).

### 4.4. First-principles Density Functional Theory Calculations

The present first-principles calculations are based on the generalized gradient approximation (GGA) to the density-functional theory [27,28]. The Perdew–Burke–Ernzerhof (PBE) form of the exchange-correlation functional was used in the present study [29]. One-electron Kohn–Sham equations are solved self-consistently by using a pseudopotential technique [30] with planewave basis sets, adopting the projected augmented plane wave (PAW) method [31], which is implemented in a scalar relativistic code of Quantum Espresso 6.3 [32,33]. The plane waves prepared with the cutoff energy of 38 Ry were used. The calculated crystal structures are based on the experimentally-determined structures, for which structural optimization for the hydrogen positions was performed. For the analysis of the density functional theory (DFT) band structure calculations, we chose the alternative setting of the unit cell with the space group of *P*2_1_*/a*. The revised lattice vectors of the unit cell are defined as *a = −(a_o_ + c_o_), b = −b_o_, c = c_o_*, where *a_o_, b_o_*, and *c_o_* indicate the original lattice vectors in *P*2_1_*/n*. We used 4 × 8 × 4 uniform *k*-point mesh (50 sampling points in the irreducible wedge of the monoclinic Brillouin zone), with a gaussian smearing method during self-consistent loops.

## Figures and Tables

**Table 1:** Room temperature conductivity, *σ*_rt_, and activation energy, *E*_a_, determined by experimental resistivity measurements, and the density functional theory (DFT) electronic band gap at the Γ point, *E*_g_, calculated using the GGA-PBE functional at various pressures.

Pressure/GPa	*σ*_rt_/S cm^−1^	*E*_a_/eV	*E*_g_ (GGA)/eV
0			0.37
3.9			0.16
5.4			0.03
5.9	1.6 × 10^−3^	0.14	
7.1			0.08
7.3	0.054	0.061	
8.2	0.31	0.063	
9.5	0.87	0.053	
9.6			0.02
10.9	1.24	0.043	
11.2			No band gap
12.9	1.06	0.053	

## Supplementary Materials

Crystallographic information files are available from the CCDC, reference numbers 1908148–1908153. These data can be obtained free of charge via http://www.ccdc.cam.ac.uk/conts/retrieving.html (or from the CCDC, 12 Union Road, Cambridge CB2 1EZ, UK; Fax: +44 1223 336033; E-mail: deposit@ccdc.cam.ac.uk).
